# Pediatric primary care provider and staff perspectives on the implementation of electronic health record-based social needs interventions: A mixed-methods study

**DOI:** 10.1017/cts.2023.585

**Published:** 2023-07-10

**Authors:** Jennifer H. LeLaurin, Jacqueline De La Cruz, Ryan P. Theis, Lindsay A. Thompson, Ji-Hyun Lee, Elizabeth A. Shenkman, Ramzi G. Salloum

**Affiliations:** 1 Department of Health Outcomes and Biomedical Informatics, College of Medicine, University of Florida, Gainesville, FL, USA; 2 Department of Pediatrics, Wake Forest School of Medicine, Winston-Salem, NC, USA; 3 Division of Quantitative Sciences, University of Florida Health Cancer Center, University of Florida, Gainesville, FL, USA; 4 Department of Biostatistics, University of Florida, Gainesville, FL, USA

**Keywords:** Implementation science, mixed methods, pediatrics, social determinants of health, electronic health record

## Abstract

**Introduction:**

Interventions to address social needs in clinical settings can improve child and family health outcomes. Electronic health record (EHR) tools are available to support these interventions but are infrequently used. This mixed-methods study sought to identify approaches for implementing social needs interventions using an existing EHR module in pediatric primary care.

**Methods:**

We conducted focus groups and interviews with providers and staff (*n* = 30) and workflow assessments (*n* = 48) at four pediatric clinics. Providers and staff completed measures assessing the acceptability, appropriateness, and feasibility of social needs interventions. The Consolidated Framework for Implementation Research guided the study. A hybrid deductive-inductive approach was used to analyze qualitative data.

**Results:**

Median scores (range 1–5) for acceptability (4.9) and appropriateness (5.0) were higher than feasibility (3.9). Perceived barriers to implementation related to duplicative processes, parent disclosure, and staffing limitations. Facilitators included the relative advantage of the EHR module compared to existing documentation practices, importance of addressing social needs, and compatibility with clinic culture and workflow. Self-administered screening was seen as inappropriate for sensitive topics. Strategies identified included providing resource lists, integrating social needs assessments with existing screening questionnaires, and reducing duplicative documentation.

**Conclusions:**

This study offers insight into the implementation of EHR-based social needs interventions and identifies strategies to promote intervention uptake. Findings highlight the need to design interventions that are feasible to implement in real-world settings. Future work should focus on integrating multiple stakeholder perspectives to inform the development of EHR tools and clinical workflows to support social needs interventions.

## Introduction

Social determinants of health (SDoH) profoundly impact child health and development [1–3]. Interventions that screen for social risks (i.e., social determinants that negatively impact health) and address patient-identified social needs for resources may improve social and health outcomes for children and families [4–8]. A number of randomized controlled trials of social needs interventions conducted in pediatric settings have demonstrated improvements in receipt of community resources, reductions in social risks, increased receipt of preventive services, reduced healthcare utilization, and improved parent and child health outcomes [9]. Despite recommendations from professional organizations, social needs are not systematically addressed in pediatric practice.10,11 Widespread adoption of these interventions is hindered by the limited body of evidence on strategies for implementing social needs interventions [7,12].

Leveraging the electronic health record (EHR) is critical to the adoption of social needs interventions in routine clinical practice. EHRs offer the opportunity to support interventions through the integration of social risk data and social needs intervention activities (e.g., referral to resources). Although EHR modules for documentation of social risks are available in many EHR systems, they are not widely used. Developing tools to support the use of these existing EHR modules can facilitate social needs screening and referral to resources, enable tracking of patients’ social needs data over time, help tailor treatment decisions, and facilitate shared decision-making [13].

There is little published guidance specific to implementing EHR-based social needs interventions in pediatric clinical care [14,15]. A handful of studies on pediatric social needs interventions using EHR tools have shown high screening rates but mixed success in identifying social needs and connecting families to resources [16–19]. This small but growing body of research offers insight into EHR-based social needs interventions in pediatrics but lacks application of implementation science frameworks, outcomes, and strategies. While one ongoing trial is formally testing implementation strategies for EHR-based social needs interventions in adults [20], there are no known comparable studies in pediatrics.

Introducing interventions in pediatric settings require consideration of unique patient-provider dynamics, clinical priorities, population characteristics, and EHR design features. Potential facilitators to the implementation of social needs interventions include a historical recognition within the field of pediatrics of the impact of family and environment on health, existing routine practices that may uncover unmet needs (e.g., nutrition screening), and additional availability of social service programs specifically for children [21,22]. Conversely, pediatric settings may face unique challenges to implementing these interventions, such as variation in relevant social risks by child age, parental concerns about child protective services (CPS), and joint custody arrangements that complicate home environment assessments. Pediatric EHR design features also make pediatric social risk data storage and access issues more complex. For example, EHR systems that allow all individuals with patient portal access to view completed questionnaires can compromise adolescent privacy and raise safety concerns [23]. In light of these added complexities related to pediatric clinical practice, more research is needed to identify tailored strategies for designing and implementing EHR-based social needs interventions in these settings.

Our study sought to address research gaps related to the implementation of EHR-based social needs interventions in pediatric settings. A primary focus of this research was the adoption of an existing SDoH EHR module. We used a convergent parallel mixed-methods design to assess multi-level factors that may impact EHR-based social needs intervention adoption and develop stakeholder-informed intervention design and implementation strategies.

## Materials and Methods

### Setting

We conducted this research as part of a larger study that aimed to identify stakeholder-informed approaches to implementing EHR-based social needs interventions in pediatric primary care. Our work examining parent perspectives on EHR-based social needs interventions is presented elsewhere [24]. In this paper, we present findings from clinical workflow assessments along with focus groups, interviews, and surveys with pediatric clinic providers and staff.

We conducted the study from February to August 2022 at four pediatric primary care clinics in a large academic health system in the southeastern United States. These clinics serve a diverse patient population and demographics vary by clinic. About half of the patient population is white, slightly less than half are insured by Medicaid, and over 70% have active patient portal accounts. All clinics use the Epic® EHR system. The study was approved by the University of Florida Institutional Review Board (#2020-03238).

**Figure 1.**
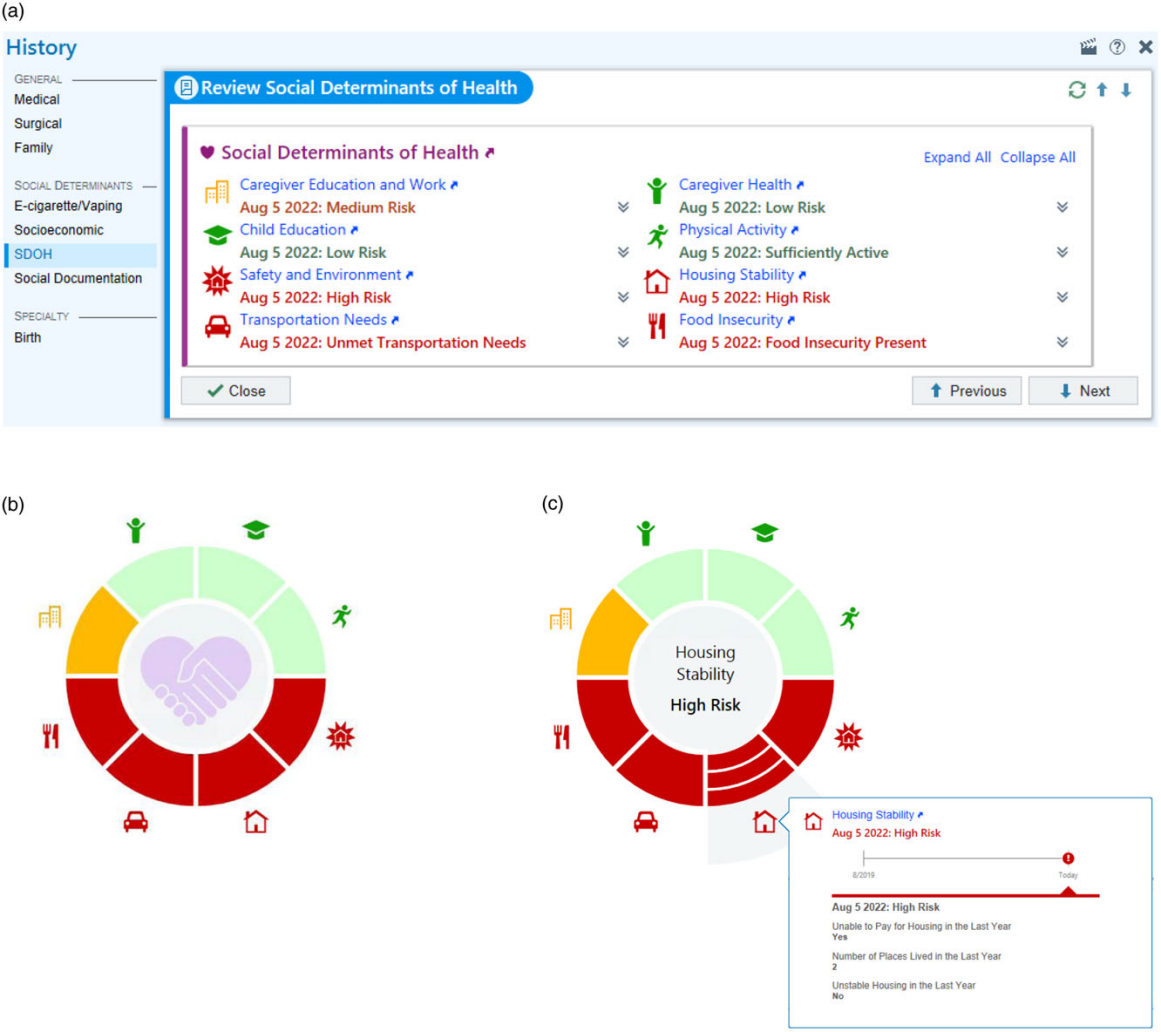
Epic® social determinants of health module for patients aged 3–10 years. ***a***) History tab displaying social determinants of health; ***b***) Wheel showing risk level by SDoH domain; ***c***) Wheel with housing domain detail pop-out.

The Epic® EHR SDoH Module contains questions assessing social risks in various domains. The module SDoH questionnaires are tailored by age and contain questions about the child, parent, and home environment (see Appendix A for sample questionnaire). This study focused on modules for patients under 11 years of age, which are intended to be answered by the parent or caregiver. Fig. 1 displays the module features, including a wheel displaying risk areas. This wheel can be incorporated into dashboards for quick assessment of patient social risks. The module also offers the capability to incorporate resource lists and referrals to address social needs.

### Conceptual Framework

We used the Consolidated Framework for Implementation Research (CFIR) to guide this work. We chose the CFIR because it can comprehensively describe the complex, multi-level factors that affect the success of evidence-based interventions in real-world settings [25]. Selected CFIR domains and constructs used in the study are presented in Table 1. Lack of conceptual clarity and inconsistent terminology limits the synthesis of implementation research. Therefore, we used the Expert Recommendations for Implementing Change (ERIC) to guide reporting of suggested implementation strategies for social needs interventions [26].

**Table 1.** Selected constructs from the CFIR [25]

Domain	Construct	Definition
Intervention characteristics	Evidence Strength & Quality	Stakeholders’ perceptions of the quality and validity of evidence supporting the belief that the intervention will have desired outcomes.
	Complexity	Perceived difficulty of implementation, reflected by duration, scope, radicalness, disruptiveness, centrality, and intricacy and number of steps required to implement.
	Relative Advantage	Stakeholders’ perception of the advantage of implementing the intervention versus an alternative solution.
Outer Setting	Patient Needs & Resources	The extent to which patient needs, as well as barriers and facilitators to meet those needs, are accurately known and prioritized by the organization.
Inner Setting	Available Resources	The level of resources dedicated for implementation and ongoing operations, including money, training, education, physical space, and time.
	Implementation Climate	The absorptive capacity for change, shared receptivity of involved individuals to an intervention, and the extent to which use of that intervention will be rewarded, supported, and expected within their organization.
Characteristics of Individuals	Self-efficacy	Individual belief in their own capabilities to execute courses of action to achieve implementation goals.

### Workflow Assessments

Workflow integration is a common challenge to the implementation of social needs interventions [14,27–29]. To better understand the context for implementation, we observed well-child visit workflows at each of the four clinics. We used a standardized patient tracking form and followed published guidance for using clinical workflow assessments to inform implementation planning [30]. During the study period, the SDoH questionnaire was not a component of standard care, but other screening questionnaires (i.e., Bright Futures health risk assessment) were assigned for all well-child visits [21]. Parents could complete these existing questionnaires before the visit via the patient portal or at the clinic on paper or tablet. Screening questionnaire completion and administration method (i.e., patient portal, paper, or tablet/kiosk) were documented when possible. Data collection procedures were designed to ensure assessments did not interfere with clinic activities.

### Focus Groups and Interviews

All providers and staff at participating pediatrics clinics were eligible and contacted for recruitment via email. We conducted focus groups at three of the clinics. Due to scheduling difficulties, individual interviews were conducted at the fourth clinic (clinic D). We included the option to conduct individual interviews in the original study protocol. We restricted interviews to physicians and residents at the fourth clinic because of low enrollment of this group in focus groups. We set a target sample size of 6–10 participants per clinic based on the anticipated adequacy of data to address the relatively narrow aims of the study [31,32], previous focus group studies on this topic [28,33], and feasibility of recruitment based on previous research in the clinics. All participants provided informed consent before study activities and received a $20 incentive for their participation.

A team member trained in qualitative methods (JHL) led focus groups and interviews. A second team member was present to assist and take notes. We held focus groups in a private room at the clinic and conducted interviews via videoconference. Sessions were audio-recorded and transcribed for analysis. At the beginning of the focus groups and interviews, we presented the Epic® SDoH module and options for screening and acting on social needs. Screening administration options presented included the patient portal, paper or tablet in the clinic waiting room, and face-to-face screening. Options for acting on social needs included provision of resource information, referral to a clinical team member (e.g., social worker), and patient navigation programs. We used the CFIR to organize the interview guide, which included questions on perceived barriers and facilitators to implementation, recommended processes and workflows, suggested EHR modifications, and necessary supports for social needs interventions implementation.

Following each focus group and interview, participants completed a brief demographic survey along with three implementation measures assessing perceived intervention acceptability, appropriateness, and feasibility [34,35]. These constructs were chosen because they may predict eventual intervention adoption and are particularly salient in the pre-implementation phase [35]. To minimize bias, participants completed the measures individually and we collected identifying information separately.

### Measures

We measured acceptability, appropriateness, and feasibility through the acceptability of intervention measure (AIM), intervention appropriateness measure (IAM), and feasibility of intervention measure (FIM) [34]. The measures assessed these constructs for social needs interventions in general, without specific reference to EHR components. Each instrument contains four items on a five-point Likert scale (one = completely disagree to five = completely agree). The AIM, IAM, and FIM have demonstrated test-retest reliability (*α* = 0.83, 0.87, and 0.88, respectively), known group validity, and structural validity [34].

### Data Analysis

For quantitative data, we performed distribution checks and generated descriptive statistics. To identify opportunities to integrate social needs interventions in well-child visits, our analysis of workflow assessment data focused on wait times at three points: waiting room (after check-in and before rooming), preexam (after rooming and before provider exam), and post-exam (after provider exam and before other activities, such as vaccinations). We examined the provider and staff AIM, IAM, and FIM results by clinic and clinic role (physician vs. other). Analysis was conducted using SPSS (IBM Corp.).

We conducted qualitative data analysis in two phases using a hybrid deductive-inductive analysis approach. In the first phase, we used rapid qualitative analysis techniques, which provide an efficient and rigorous approach that is well-suited for implementation research [36]. We first developed a summary template with domains based on the CFIR and interview guide. The template also included fields for content that fell outside of these domains. We used the template to extract relevant content from each transcript and categorize it by domain. Content included summarized participant statements and direct quotes. We piloted the template with two transcripts before applying it to all transcripts. One team member (JHL) used the template to extract data from all transcripts and a second team member (JDLC) performed audits on 30% of the transcripts to identify any missing, irrelevant, or miscategorized content.

The first phase of analysis resulted in a clinic-by-domain matrix containing summarized data and quotes relevant to the study aims. In the second phase, we used a general inductive analysis approach [37], consisting of iteratively coding the summarized data in the matrix and organizing codes into categories or themes. First, we developed a preliminary codebook informed by observations from the first phase of analysis. Two team members then independently coded the summarized data from one focus group and met to compare coding, reconcile differences, and revise the codebook as needed. We repeated this process for two focus groups and two interviews. One team member coded data from the remaining focus group and interviews independently. No new codes were identified after data from three focus groups and two interviews were coded. We constructed an aggregate summary of findings from the clinic D interviews to examine results by clinic. We organized themes relating to implementation determinants (e.g., barriers and facilitators) by the CFIR and implementation strategies by the ERIC taxonomy [26]. An audit trail documenting all coding decisions and codebook changes was maintained throughout the analysis process.

### Data Integration

We used a merging approach to combine workflow, survey, and qualitative data [38]. We constructed side-by-side joint displays (i.e., matrices) to visually present corresponding quantitative and qualitative data and triangulate findings. We reviewed workflow analysis findings in conjunction with qualitative data related to clinic processes and workflows to assess the feasibility of integrating social needs interventions in each clinic. To obtain a deeper understanding of results from the implementation measures (AIM, IAM, FIM), we examined quantitative results from the overall sample in conjunction with themes identified through qualitative analysis. We mapped themes to relevant implementation outcomes and assigned a valence to each theme to represent a positive, negative, or mixed impact on the implementation outcome.

## Results

### Workflow Assessment

We observed 12 patient visits at each clinic (n = 48). Wait times for workflow components are presented in Table 2. It was not possible to systematically assess the completion of paper-based questionnaires because this would have interfered with clinical activities. A review of screening questionnaires captured in the EHR (i.e., completed via the patient portal or EHR-linked tablet) showed that of 10 patients with submitted questionnaires, five completed all questions and five partially completed the questionnaires.

**Table 2.** Patient wait times by workflow component and clinic (*n* = 48)

	Patient wait time (minutes)			
Workflow component	Clinic A	Clinic B	Clinic C	Clinic D
Waiting room				
Mean (SD)	10.2 (11.0)	4.2 (4.8)	6.1 (5.3)	3.9 (1.7)
Median (min-max)	5.5 (1.0–39.0)	3.0 (1.0–18.0)	3.5 (2.0–19.0)	3.5 (2.0–7.0)
Pre-Exam				
Mean (SD)	9.4 (6.2)	10.8 (8.4)	7.1 (7.3)	7.5 (6.5)
Median (min-max)	9.0 (2.0–21.0)	7.5 (2.0–29.0)	4.0 (0.0–24.0)	7.0 (0.0–21.0)
Post-Exam				
Mean (SD)	6.9 (8.9)	2.7 (2.9)	2.0 (2.6)	1.6 (4.0)
Median (min-max)	3.5 (0.0–28.0)	1.5 (0.0–8.0)	1.0 (0.0–8.0)	0.0 (0.0–14.0)

Provider and Staff Focus Groups, Interviews, and Surveys

A total of 30 providers and clinic staff participated in three focus groups (clinics A, B, and C) and six interviews (clinic D). Provider and staff characteristics are presented in Table 3. Physicians and advanced practice providers comprised half the sample. All participants were female and most identified as white and non-Hispanic. Four participants also held clinic leadership roles (i.e., clinic directors and clinic managers). The all-female sample is consistent with the clinics’ provider and staff demographics.

**Table 3.** Provider and staff characteristics (*n* = 30)

Characteristic	*n*	%
Clinic		
Clinic A	7	23.3
Clinic B	10	33.3
Clinic C	7	23.3
Clinic D	6	20.0
Gender		
Female	30	100.0
Race		
White/Caucasian	22	73.3
Black/African American	5	16.7
Multiple	2	6.7
Other	1	3.3
Ethnicity		
Hispanic	5	16.7
Non-Hispanic	25	83.3
Clinic Role		
Physician^a^	9	30.0
Advanced practice provider^b^	6	20.0
Registered nurse	9	30.0
Medical assistant	4	13.3
Administrative staff	2	6.7
Clinic leadership^c^	4	13.3

a Includes residents.

b Includes nurse practitioners and physician assistants.

c Clinic leadership (i.e., clinic directors and clinic managers) also hold other roles, therefore totals exceed 100%.

### Surveys

Table 4 displays descriptive statistics for the perceived AIM, IAM, and FIM by clinic and clinic role. Most participants had total scores over 4.0 (indicating “agree” or “completely agree”) for acceptability (*n* = 25, 83.3%) and appropriateness (*n* = 27, 90.0%), with lower rates observed for feasibility (*n* = 15, 50%). The overall mean and median FIM scores were lower than the AIM and IAM scores. This pattern was consistent across clinics and clinic roles. Internal consistency was excellent for the AIM (*α* = 0.91) and IAM (*α* = 0.96) and good for the FIM (*α* = 0.85).

**Table 4.** Perceived intervention acceptability, appropriateness, and feasibility by clinic and clinic role (*n* = 30)^a,b^

Clinic/role	AIM	IAM	FIM
All participants			
Mean (SD)	4.6 (0.6)	4.6 (0.6)	4.0 (0.7)
Median (min-max)	4.9 (3.3–5.0)	5.0 (3.0–5.0)	3.9 (2.5–5.0)
Clinic (mean, SD)			
Clinic A	4.4 (0.2)	4.6 (0.3)	4.1 (0.8)
Clinic B	4.7 (0.5)	4.6 (0.5)	3.9 (0.8)
Clinic C	4.3 (0.7)	4.3 (0.7)	3.9 (0.5)
Clinic D	5.0 (0.1)	4.9 (0.2)	4.2 (0.5)
Clinic Role (mean, SD)			
Physician	4.8 (0.4)	4.8 (0.4)	4.0 (0.5)
Advanced practice provider	4.5 (0.5)	4.4 (0.5)	3.4 (0.4)
Registered nurse	4.5 (0.7)	4.5 (0.7)	4.1 (0.5)
Medical assistant	4.3 (0.8)	4.5 (1.0)	4.1 (1.1)
Administrative staff	5.0 (0.0)	5.0 (0.0)	5.0 (0.0)
Clinic leadership	4.4 (0.7)	4.5 (0.6)	4.2 (0.8)

a AIM = acceptability of Intervention measure; IAM = intervention appropriateness measure (IAM); FIM = feasibility of intervention measure.

b Possible scores range 1–5.

### Focus Groups

#### Qualitative themes by the CFIR domains

Table 5 presents themes from the focus groups and surveys organized by CFIR constructs, along with illustrative quotes and corresponding implementation outcomes.

**Table 5.** Focus group and interview themes by the CFIR constructs

CFIR construct	Theme	Illustrative quote
**Intervention characteristics**		
Evidence Strength & Quality	SDoH strongly impact patient and family health	“They come in with a lot of symptoms, but I think the diagnosis is a lot of the time one of these determinants.” [Resident, Clinic D]
	Screening for and addressing social needs can improve healthcare by enhancing provider understanding and meeting family social needs	“I found out a kid was homeless one day and that kind of made sense, all of a sudden, for a bunch of different reasons for why visits weren't going that well.” [Physician, Clinic D]
Complexity	Module items are redundant with currently used screening tools and processes but offer opportunities to consolidate data	“I ask every patient, “Do you wear your seatbelt? Do you have transportation? Is there alcohol at home?” But I have to type that in my notes, so I would never type that and then go do this afterwards as well.” [Advanced Practice Provider, Clinic B]
Relative Advantage	The EHR module can improve existing processes to address social needs	“I think that it would be a great way of assessing more of those aspects than we would otherwise.” [Resident, Clinic D]
**Outer setting**		
Patient Needs and Resources	Perception that parents do not disclose social needs due to discomfort, lack of trust, or fear of negative outcomes	“I have a patient that has been my patient for seven years, so I felt like I knew them very well. And at one point they lived in their car for three months and I had no idea until after they had a place, because she was like, “I didn't want them to take my kid away.” She didn't ask for help, she didn't tell anybody, she just figured it out and then told me after.” [Advanced Practice Provider, Clinic B]
Patient Needs and Resources	Participating in social needs interventions is perceived as burdensome for parents	“[Parents are] more focused on getting their child in and not waiting a long time, especially with the families who catch rides into the clinic. They have to ride the bus or they have to get a cab voucher…they're saying “I have to go to work, I cannot be here, I'm going to get in trouble at my job.”” [Registered Nurse, Clinic A]
Patient Needs and Resources	Lack of available resources, services, or personnel is a barrier to social needs interventions.	“Well how do we help them? We refer them to social work sometimes … I do not know how much is that really helping them.” [Advanced Practice Provider, Clinic B]
Patient Needs and Resources	Access to technology and rates of patient portal use affect the reach of pre-visit screening	“We have the highest efficiency with patient portal utilization within our organizations. For us, we have the ability to obtain information prior to them even walking into the building so it really wouldn't interrupt our clinic flow.” [Administrative Staff, Clinic C]
**Inner setting**		
Available Resources	Implementation of social needs interventions is difficult due to time, staffing, and workload limitations	“If we are implementing something, but we do not have the infrastructure necessary to provide resources, or have consultants to be able to reach out to, we're doing our patients a disservice by not being able to connect them with the resources.” [Resident, Clinic D]
Compatibility	Social needs interventions align with clinic values, culture, and processes	“[The residents] teach me. And they say “Oh I looked at this” and I’m like, “Wait you did this? What is that? Okay, let me see if I should do that too.” You know part of being in academic medicine is it keeps me on my toes, so I don't become complacent.” [Physician, Clinic D]
**Characteristics of individuals**		
Self-efficacy	Providers and staff feel better equipped to screen for social risks than to address social needs	“I feel comfortable talking to patients about homelessness but I do not know where the shelters are or how to get there.” [Resident, Clinic D]

In addition to discussing the potential barriers and facilitators to implementing social needs interventions using the EHR module, participants also related their experiences identifying social risks and addressing social needs through referrals to social workers in the course of existing care practices. Themes by the CFIR domains are described below.

#### Intervention Characteristics

In relation to the CFIR construct of evidence strength and quality, participants described both the impact of social risks on patient outcomes and how interventions to address social needs could result in improved care and outcomes. They shared case examples illustrating how social risks impact patient and family health, including connections between neighborhood safety and physical activity, food insecurity and nutrition, and transportation and healthcare access. Participants viewed social needs interventions as an opportunity to not only directly address family needs but also as a method to improve healthcare by offering a more holistic view of patients, enhancing provider understanding of the causes of health problems, and increasing patient satisfaction.

Concerning intervention complexity, providers and staff noted some SDoH module domains were duplicative with existing questionnaires and standard well-child visit documentation. While this meant using the module in its current form would add complexity to visits and documentation, participants felt the module could reduce the complexity of current screening practices if data entry was integrated (e.g., cross-population of data). Participants expressed appreciation for the potential opportunity to consolidate existing documentation.

Although providers and staff described screening for social risks and addressing some social needs in their current practice, they felt using the module could offer advantages over these existing processes. Participants described the module domains as “more comprehensive” and the questions as “more specific” than existing questionnaires and screening procedures. Participants also highlighted the helpfulness of the SDoH wheel visual in quickly assessing risk factors.

#### Outer Setting

Anticipated lack of patient disclosure was identified as a barrier to social needs interventions in nearly all interviews (*n* = 5) and all three focus groups. Participants felt that parents would not share needs due to embarrassment, lack of trust, and fear of negative outcomes. They often discussed parents’ fear of the CPS and losing custody of their children. Providers and staff emphasized the need to build trust with families but also noted this was difficult when families were unable to consistently see the same provider.

Participants described barriers related to the burden interventions may place on families. Providers did not feel interventions that would lengthen visit times would be feasible for parents who use public transportation or struggle to take time off of work. Participants felt parents with social needs may not complete the SDoH module questionnaire due to lack of time, competing demands, and stress. They noted families often did not complete other screening questionnaires administered through the patient portal and worried an additional screening would exacerbate questionnaire fatigue among parents.

Providers and staff identified a lack of available resources as a barrier to the effectiveness of social needs interventions. Some expressed uncertainty about the effectiveness of social work referrals due to the limited availability of community services. They gave examples of successfully connecting families with food insecurity to resources (e.g., federally-funded food assistance programs), but shared how addressing other social risks, such as housing instability and mental health conditions among parents, was a challenge. Additional barriers included eligibility requirements and limits on service use. Participants also noted disparities in resource availability for rural residents and Spanish-speaking families.

Patient portal use and technology access were seen as both barriers and facilitators to the reach of social needs interventions. Descriptions of patient portal activation and use varied significantly by clinic. A staff member noted the high rates of patient portal use at clinic C as a facilitator, while a physician at clinic B stated “maybe 10%” of her families completed existing pre-visit screening via the portal. Participants attributed low questionnaire completion to factors including higher stress and competing demands among low-income families. While some participants cited lack of access to technology as a barrier to patient portal screening, other participants noted high rates of smartphone ownership, even among families of low socioeconomic status. They saw this as an opportunity to reach more families and conveniently provide resources.

#### Inner Setting

Concerning the CFIR inner setting construct of available resources, participants raised concerns about the capacity of clinics to implement social needs interventions. Providers and staff cautioned against screening for social risks without available resources to address families’ needs. They consistently identified a lack of time as a barrier to screening for social risks. They also noted patients with social needs often had other issues to address during the visit. Participants also conveyed concerns related to increasing workloads, especially for pediatric social workers.

Providers and staff felt social needs interventions were compatible with the goals of pediatric primary care, organizational culture, and clinic processes. In clinic D, physicians and residents described a learning culture that encouraged the introduction of new practices through a “self-perpetuating” cycle of attending-resident knowledge exchange as conducive to implementing new practices. Participants also discussed how addressing social needs fits within the well-child visit model and social risk questions could be integrated with existing screening processes.

#### Characteristics of Individuals

Participants expressed a desire to help address social needs but had low self-efficacy due to lack of knowledge and resources. Providers described feeling comfortable screening for social risks but lacked confidence in effectively addressing identified needs. They felt if they had appropriate training or resources, they could help families with less complex needs. As one participant stated, “I wonder how many of those calls we sent to [the social worker] are really something we could answer.”

### Intervention delivery

Opinions differed on whether in-person or self-administered screening would encourage parents to disclose social risks. Some providers and staff felt a self-administered screening would mitigate parent discomfort and embarrassment associated with disclosing this information. Others felt parents would be more open with verbal screening, especially for sensitive needs. When discussing physical or sexual abuse, one physician stated, “if they’re going to tell me, they’re going to tell me in the clinic setting.” Participants felt verbal screening for sensitive social risks was most appropriate, while other needs could be assessed via self-administered questionnaires.

Participants from clinic C felt patient portal administration would work well for their setting given high portal activation rates; however, participants from the other clinics doubted most parents with social needs would complete the questionnaire before the visit. Lack of time and privacy were cited as barriers to offering face-to-face screening in clinic waiting rooms. Some participants felt a nurse or medical assistant might be able to ask parents questions before the exam, but only if the questionnaire was reduced to two or three questions. Participants also noted that while completing screening questionnaires in the waiting room was typical, often parents did not have time to complete the full questionnaire.

All clinics relied heavily on social work referrals to address family social needs. Participants expressed concerns that increased social risk screening could result in an unmanageable workload for social workers. They felt it would be helpful to provide families with information on community resources to address more general or common social needs via brochures, texted links, patient portal messages, quick response (QR) codes, or inclusion in visit summary paperwork. They were also supportive of stocking brochures and other resources in the clinic waiting and exam rooms to provide help to parents who were unlikely to disclose social risks during screening. The need for a centralized location of online resources was also noted. One challenge identified with these methods was maintaining up-to-date resource lists.

### Implementation strategies

Suggested strategies for supporting the implementation of social needs interventions organized by ERIC domain are presented in Table 6. Participants offered specific suggestions to improve the uptake of the module through EHR modifications, including increasing the visibility of the wheel, populating module data from other questionnaires and data sources, and including social risk screening in visit note templates. Several adaptations were proposed, including integrating social risk assessments in existing questionnaires, reducing the number of risks in the screening, and conducting social risk screening and intervention outside of the well-child visit. While some participants expressed support for conducting post-visit assessments, others noted it was important for the provider to assess and address social needs during the visit.

**Table 6.** Suggested implementation strategies by ERIC [26] domain

ERIC domain	Implementation strategy	Suggested strategies
Change Record Systems	EHR modifications	Automatically populate the module with responses from existing questionnaires and visit documentation (e.g., social history) Display SDoH wheel in commonly used tabs Incorporate EHR module in note templates Use “smart phrases” to quickly generate resource lists Reminders and alerts Reduce clicks
Adapt and Tailor to the Context	Streamline screening	Integrate social risk questions in existing screening tools and processes
	Revise questionnaire	Reduce number of questions Tailor administration method by question sensitivity
	Separate from well-child visits	Conduct social risk screening and discussion post-visit
Engage Consumers	Educate and support parents	Raise awareness of clinic as a source for social needs support Communicate purpose of screening Promote patient portal use
	Build trust	Schedule families with a consistent provider Hire more Spanish-speaking personnel and translate questionnaires
Support Clinicians	Engage clinical team members	Have nurses and medical assistants administer screening Use health coaches to assess and address needs Provide resource lists instead of referring to social work for certain social needs
	Improve staffing	Hire more social workers Hire more Spanish-speaking providers and staff Engage social work students
Train and Educate Stakeholders	Equip providers and staff with knowledge and resources to address social needs	On-site EHR module training Incorporate how to address social needs in medical education Target residents for training Present at grand rounds

Engaging families through education, support, and trust-building was noted as a priority. Participants noted some families were not consistently scheduled with the same provider and improving provider continuity would help build trust. Involving clinical team members in social needs interventions was generally seen as feasible for less sensitive needs. One provider cautioned against duplicative screening for sensitive issues, stating “if there is an issue, you’re just re-bringing it up in the visit, and now they've had to talk about their homelessness to multiple people.” Participants noted the need to hire more social workers as well as Spanish-speaking providers and staff. Providers and residents felt hands-on EHR training delivered at the clinic would be helpful. Several participants noted that residents were a good target for training.

### Data Integration

The views of providers and staff that additional screening would be difficult due to time limitations were supported by workflow observation findings. Several proposed strategies addressed these workflow challenges, including EHR modifications, reducing the number of questionnaire items, and streamlining items with existing questionnaires. Providers and staff gave relatively high ratings for social needs intervention acceptability and appropriateness, while feasibility ratings were somewhat lower. In focus groups and interviews, participants viewed barriers such as lack of patient disclosure, limited community resources, parent burden, and clinical capacity as limiting the feasibility of the intervention. On the other hand, most facilitators related to intervention acceptability and appropriateness. Although survey data indicated high perceived acceptability and appropriateness, qualitative findings provided a more nuanced view of the interplay of factors affecting these outcomes. Notably, all themes related to the CFIR construct of patient needs and resources were seen as negatively impacting implementation outcomes except for technology use, which had a mixed impact.

## Discussion

This study elicited stakeholder perspectives on the implementation of EHR-based social needs interventions in pediatric primary care. Providers and staff generally viewed social needs interventions as acceptable and appropriate for this clinical setting and proposed numerous strategies to address implementation barriers. Previous publications have detailed efforts to implement social needs interventions; however, with a few exceptions, [20,39,40] these publications lack application of implementation science frameworks, use of established implementation outcomes, or explicit description of implementation strategies. The application of the CFIR, administration of validated implementation measures, and use of the ERIC taxonomy of implementation strategies in this study is a step toward standardizing the conceptualization and reporting of implementation determinants, outcomes, and strategies in social needs research.

Results from the provider and staff implementation surveys indicated relatively high levels of perceived acceptability and appropriateness, but lower levels of perceived feasibility. A previous study using the same instruments in a post-implementation assessment of a social needs intervention for medically complex children reported a similar pattern of results [40.] Our use of a mixed-methods approach provides further insight into the quantitative findings. Providers and staff stressed the difficulty of introducing additional screening in time-sensitive workflows, a sentiment that was supported by findings from clinical workflow assessments. One study of a pediatric primary care social needs intervention found a negligible impact on visit time; however, over 25% of potentially eligible families were excluded due to insufficient time to conduct the screening in the waiting room [4]. Workflow challenges are consistently cited as a barrier to implementing social needs interventions in clinical settings [14,27,29], emphasizing the need for additional research on tailoring workflows to support implementation of new clinical practices.

Although completion of social risk screening through the patient portal reduces clinical workflow impact, participants expressed differing views on parent uptake of portal-based screening among clinics. These views were influenced both by clinic portal activation rates and patient population characteristics. This finding demonstrates the need for multiple screening administration options and tailored strategies by setting, especially in light of the potential for patient portal-based interventions to exacerbate existing disparities [41,42].

Some providers and staff felt self-administered questionnaires would mitigate potential embarrassment and encourage patient disclosure; however, others felt screening methods should be contingent on the sensitivity of the topic. While some evidence indicates self-administered screening may elicit more disclosure of social risks [43], previous efforts to implement social risk screening have omitted sensitive items deemed more appropriate in a face-to-face encounter from screening instruments [15,44]. Further, including questions related to mandatory reporting requirements (e.g., child abuse or neglect) on a self-administered questionnaire introduces risk of liability if the responses are not reviewed and addressed by a provider. Some settings have chosen to use more general language on screening instruments to address this issue [44]. In light of recommendations from providers and staff in this study to reduce the number of items on the social risk screening questionnaire, moving sensitive items to verbal provider screening could address multiple implementation challenges.

Providers and staff in this study generally felt comfortable screening for social risks but lacked the knowledge and resources to address social needs. Equipping clinics with resource lists was seen as a feasible strategy to help families, especially for parents reluctant to engage with social work services. Research on the effectiveness of information provision versus patient navigation is mixed [6,45,46]. One randomized controlled trial found patient navigation and provision of resource lists in pediatric urgent care equally effective in addressing social needs and improving child health [45], though a secondary analysis of study data found patient navigation was associated with reductions in subsequent pediatric hospitalization rates [46]. While more research is needed on this issue, offering resource lists is a low-cost strategy that can be integrated into EHR modules and may increase the reach of social needs interventions in limited-capacity settings.

In response to calls for more research on leveraging the EHR to support social needs interventions [20], we elicited stakeholder feedback on specific EHR modifications to improve the existing Epic® SDoH module. Participants in this study described how some EHR module domains were duplicative with data collected from existing pediatric screening instruments and provider documentation in visit notes. Participants saw this as an opportunity to streamline screening questionnaires and cross-populate data in the EHR, addressing concerns cited in previous studies that scattered SDoH documentation would result in a fragmented view of patients [14]. For example, all well-child visits at the clinics are assigned an EHR-integrated health risk assessment that screens for some social risks also included in the SDoH module (e.g., food insecurity). Integration of these questionnaires would allow structured EHR documentation of patient SDoH with minimal impact on patient burden and clinical workload. Participants also suggested embedding the SDoH module items in existing well-child encounter note templates, a method that has shown success in other settings [17,47]. While these strategies are promising, the deployment of these modifications requires leadership buy-in, healthcare system prioritization, and information technology (IT) resource allocation.

In addition to EHR modifications, providers and staff offered multiple strategies to support implementation of social needs interventions. Participants suggested some strategies successfully employed in other settings, such as adapting screening questionnaires, education and training, and tailored workflows [14,27,44]. Providers and staff also proposed strategies related to engaging families through education, support, and trust-building, which are less frequently described in the literature. Other suggestions were reflective of the academic medical center setting, such as targeting residents and incorporating social needs intervention training into medical education.

This study has several limitations. Due to the small sample size, only descriptive statistics were generated for quantitative data and results should be viewed as preliminary. Future quantitative studies using larger sample sizes should be conducted to assess generalizability and explore differences between clinical groups. As previously noted, some of the findings may be specific to academic health systems and not transferrable to other settings. Another limitation of the study relates to the sampling approach. To compensate for the suboptimal engagement of physicians in clinic focus groups interviews were conducted exclusively with physicians and residents at one clinic. This approach elicited valuable insight on topics not fully explored in focus groups; however, we did not obtain the perspectives of clinical support staff at this clinic. Further, while the focus groups at three of the clinics likely offered greater depth of information and brainstorming of ideas, the interview format used in the fourth clinic may have reduced social desirability bias and non-disclosure. Finally, we do not present the perspectives of patients and families in this paper. Future work should focus on identifying areas of overlap in the preferences of both stakeholder groups to inform intervention design and implementation strategies.

## Conclusion

This study offers valuable insight into the implementation of EHR-based social needs interventions in pediatric settings. Although providers and staff are receptive to EHR-based social needs interventions, significant implementation challenges exist. Our work identifies specific strategies for overcoming these barriers and promoting the uptake of these interventions in real-world settings. Future work should focus on integrating provider and staff perspectives with feedback from other stakeholders (e.g., families, IT professionals) to develop EHR tools and clinical workflows for social needs interventions that are both feasible to implement and acceptable to families.

## Supplementary material

For supplementary material accompanying this paper visit https://doi.org/10.1017/cts.2023.585. click here to view supplementary material
