# Supplementary material for: Pediatric primary care provider and staff perspectives on the implementation of electronic health record-based social needs interventions: A mixed-methods study
Source: J Clin Transl Sci. 2023 Jul 10;7(1):e160. doi: 10.1017/cts.2023.585 (PMC10388413; doi:10.1017/cts.2023.585)
Supplement: Supplementary file 1 [file S205986612300585Xsup001.docx]

Appendix A

Epic^®^ Social Determinants of Health Questionnaire

Physical Activity

1. On average, how many days per week do you engage in moderate to strenuous exercise (like a brisk walk)?
2. On average, how many minutes do you engage in exercise at this level?

Food Insecurity

1. Within the past 12 months, you worried that your food would run out before you got money to buy more:

- Never true
- Sometimes true
- Often true
- Decline

1. Within the past 12 months, the food you bought just didn't last and you didn't have money to get more:

- Never true
- Sometimes true
- Often true
- Decline

Transportation Needs

1. In the past 12 months, has lack of transportation kept you from medical appointments or from getting medications?

- Yes
- No
- Decline

1. In the past 12 months, has lack of transportation kept you from meetings, work, or getting things done for daily living?

- Yes
- No
- Decline

Housing

1. In the last 12 months, was there a time when you were not able to pay the mortgage or rent on time?

- Yes
- No
- Decline

1. In the last 12 months, how many places have you lived?
2. In the last 12 months, was there a time when you did not have a steady place to sleep or slept in a shelter (including now)?

- Yes
- No
- Decline

Caregiver Education and Work

1. Do you have a high school degree?
   - Yes
   - No
   - Decline to answer
2. Do you ever need help reading hospital materials?
   - Yes
   - No
   - Decline

Caregiver Health

1. Over the past two weeks, how often have you felt little interest or pleasure in doing things?
   - Not at all
   - Several days
   - More than half the days
   - Nearly every day
   - Decline
2. Over the past two weeks have you been bothered by feeling down, depressed, or hopeless?
   - Not at all
   - Several days
   - More than half the days
   - Nearly every day
   - Decline
3. Does anyone in your home have a problem with alcohol, marijuana, or other substances?
   - Yes
   - No
   - Decline

Child Education

1. Is your child in Head Start, preschool, or early childhood enrichment?
   - Yes
   - No
   - Decline
   - Not applicable
2. Is your child doing well in school? Are they getting the help to learn what they need?
   - Yes
   - No
   - Decline
3. Do you read to your child every night?
   - Yes
   - No
   - Decline

Safety and Environment

1. Do you worry that your child may have been physically abused?
   - Yes
   - No
   - Decline
2. Do you worry that your child may have been sexually abused?
   - Yes
   - No
   - Decline
3. Are there any guns kept in or around your home or where your child spends time?
   - Yes
   - No
   - Decline
